# Trust-Building Strategies of Fundraising Consultants in Chinese Medical Crowdfunding Platforms: Qualitative Study

**DOI:** 10.2196/80299

**Published:** 2025-11-19

**Authors:** Qiong Li, Jianyuan Huang, Yiting Tan, Lina Du, Tifeng Liu

**Affiliations:** 1 College of Public Administration, Hohai University, Nanjing, China; 2 International Cooperation Office, Jiangsu Province Hospital, No.300, Guangzhou Road, Nanjing, 210009, China, 86 02568306427

**Keywords:** medical crowdfunding, fundraising, patients and families, trust, medical assistance, sustainable development, qualitative research, interviews

## Abstract

**Background:**

In recent years, Chinese internet-based medical crowdfunding platforms have faced widespread public skepticism and a macrolevel climate of trust crisis, which has inevitably exacerbated distrust among patients and their families toward these platforms. In this context, fundraising consultants, as frontline executors who directly engage with patient families, play a pivotal role in the operational models of mainstream platforms in China, serving as a critical trust bridge between platforms and potential help-seekers. The core responsibilities of fundraising consultants extend beyond explaining platform rules to resolving the doubts of patients and their families through interpersonal interaction, building trust, and effectively mobilizing and assisting them in launching crowdfunding campaigns.

**Objective:**

This study aims to explore the core strategies used by fundraising consultants to gain the trust of patients and their families and to deeply analyze the impact of these strategies on the sustainable development of medical crowdfunding platforms—areas that have not been explored in existing literature.

**Methods:**

A phenomenological qualitative research design involving in-depth semistructured interviews was chosen. Purposeful sampling was used to obtain a heterogeneous sample, with 16 fundraising consultants from 4 Chinese internet medical crowdfunding platforms participating in the study. After reaching data saturation, 2 additional consultants were interviewed. Recruitment was terminated when no new themes emerged. Data analysis was based on the Colaizzi method.

**Results:**

In total, 16 fundraising consultants from 4 Chinese medical crowdfunding platforms participated in the study. Three main themes and nine subthemes emerged: (1) establishing initial communication (using scripted opening techniques to initiate conversations and expressing empathy and care), (2) identifying doubts (authenticity of identity, standardization of fund operations, and security of personal information), and (3) addressing doubts (presenting valid credentials, leveraging endorsements from medical institutions, displaying successful cases, and promising additional conditions).

**Conclusions:**

The findings reveal that the trust-building strategies of fundraising consultants exhibit a dualistic divide between positive and negative approaches. Positive strategies effectively resolve patients’ trust barriers, accumulate a long-term reputation for platforms, and form a positive driving force for healthy development. Negative strategies, while potentially increasing crowdfunding initiation in the short term, severely damage platform credibility in the long run, becoming hidden dangers that hinder sustainable development. Based on these findings, this study proposes optimization suggestions to enhance platform social trust, promote the standardized and sustainable development of medical crowdfunding platforms, and construct a reliable social support network for patients in need.

## Introduction

In recent years, medical crowdfunding platforms enabled by internet technology have emerged and rapidly developed in multiple countries. Such platforms raise medical expenses for individuals from dispersed audiences [1], providing an alternative fundraising channel that plays a critical role in reducing out-of-pocket medical costs [2]. In China, since their rise around 2014, medical crowdfunding platforms have cumulatively raised over 100 billion yuan (approximately US $14.1 billion) for major illness relief, nearly equivalent to the total central government medical assistance allocations from 2018 to 2021 [3]. Their social influence is deeply embedded in the landscape of medical resource allocation, highlighting the significance of in-depth research on these platforms.

International academic studies on medical crowdfunding primarily focus on 3 areas. First, factors influencing crowdfunding success. Existing literature systematically explores the roles of multidimensional factors, such as platforms, fundraisers, and donors [4], aiming to identify key determinants of campaign success. At the platform level, as the core hub connecting donors and fundraisers, it plays a vital role in resource matching and information transmission [5]. Studies confirm that basic platform functions like user-friendly interface design and information transparency [6], as well as dynamic display elements, such as image quantity and project update frequency [7], are significantly correlated with fundraising effectiveness. At the fundraiser level, patient characteristics (age, region, and economic status of residence) [8], disease type, treatment stage, and diagnostic certificates [9] effectively enhance public attention and donation willingness. Additionally, fundraisers’ narrative strategies and story texts [10], along with extensive social network mobilization [6], significantly expand project exposure and fundraising scale. From the donor perspective, intrinsic factors, such as altruism, compassion, [11], and preferences [12] are core motivations for donation behavior. For example, a study suggests that individual donors are more inclined to choose donation amounts with multiple characteristics, such as 5, 10, 20, and 50 [12]. The group that has experienced donating often shows a stronger willingness to repeat donations due to their higher trust in crowdfunding models.

Second, while providing critical medical assistance, medical crowdfunding platforms have spawned significant fraud risks, shifting research focus to ethical risks. The model’s low entry barrier, physical distance between fundraisers and donors, and high anonymity in certain links pose substantial integrity challenges [13]. Take GoFundMe, one of the world’s largest crowdfunding platforms, as an example: it has raised over US $5 billion since 2010, but the ensuing trust crisis has drawn widespread attention [14]. Research has identified 4 typical fraud categories, such as fabricating or exaggerating one’s own illness, falsifying others’ illnesses, identity impersonation, and misappropriation of donations [15]. These actions not only directly erode donors’ trust but also undermine public confidence in industry regulatory effectiveness, leading to severe trust crises.

Third, the complex impacts and negative consequences of medical crowdfunding continue to grow [16], becoming a key topic in scholarly discussions. Specifically, while medical crowdfunding offers flexible financial solutions, its potential fairness crisis cannot be ignored [17]. Studies indicate that its resource allocation mechanism exacerbates social inequality [18]. Relatively affluent groups with abundant social capital, digital literacy, and media influence leverage platform rules through crafted narratives and social network dissemination to secure more medical support [19]. In contrast, vulnerable groups, constrained by the digital divide and lacking technical and social mobilization resources, often fail to bring their medical needs to public attention. This differential outcome stems from the inherent limitations of personalized charity. When public medical assistance decisions rely on emotional preferences and subjective values [16], resource allocation deviates from institutionalized, universal frameworks and becomes dependent on individual social capital. In the long run, medical crowdfunding may not only fail to bridge health resource gaps but also solidify social stratification in medical accessibility [20], systematically impacting the fairness and sustainability of public medical security systems.

In China, despite attracting public attention and support [21] and alleviating patients’ economic burdens, medical crowdfunding platforms face multiple challenges [22]. First, literature shows a contradiction between commercialization and public welfare. To increase market share, many platforms deploy fundraising consultants for large-scale hospital marketing [23], linking business volume directly to consultants’ performance [24], which frequently triggers negative publicity about excessive marketing and potential fraud [25]. Second, systemic loopholes exist, including questionable information authenticity, insufficient transparency, perfunctory review and regulation, and privacy breaches [26]. These issues not only harm applicants but also erode donors’ basic trust. Finally, scholars emphasize that most platforms do not disclose key information (eg, matching between raised funds and actual needs), raising concerns about irregular fund use and untraceable flows [27]. The public’s inability to verify fund usage triggers doubts about platform legitimacy and operational purposes [28], progressively weakening donation willingness and hindering industry sustainability. Regarding the above issues, some scholars point out that the platform’s predicament stems from a dual reason. From an internal perspective, medical crowdfunding platforms are mostly commercial organizations that pursue profit maximization. They value the number of users, funding, and financing potential, placing public welfare value in a secondary position [29]. This business orientation gives it a tendency to relax information screening and lower review thresholds to attract users [30]. At the same time, the platform also has obvious shortcomings in information governance technology and management capabilities [31]. From the perspective of external factors, China’s relevant legal construction lags behind, and there is a lack of sound legal regulation and effective supervision on the professional ethics and fund flow of fundraising consultants [32].

It is noteworthy that although public skepticism regarding the authenticity of crowdfunding stories primarily arises from the donor’s perspective, this macrolevel climate of trust crisis [33] inevitably exacerbates distrust among patients and their families toward the platforms. Within this complex context, fundraising consultants—serving as frontline executors who interact directly with patient families—play a vital role in the operational model of mainstream platforms in China, acting as a critical trust bridge between platforms and potential help-seekers. For internet-based medical crowdfunding—highly dependent on trust [34]—consultants’ core responsibilities extend beyond explaining platform rules to resolving patient and family doubts through interpersonal interaction, building trust, and mobilizing them to launch campaigns. Therefore, investigating the trust-building strategies used by fundraising consultants is essential for understanding how platforms activate user participation, maintain daily operations, and achieve sustainable development. However, medical crowdfunding research remains in its infancy [35]. Existing studies, while offering macrolevel insights into operations and institutional flaws, overlook the deep value of microlevel interactions between platforms and patient families [36], particularly the lack of inquiry into consultants’ trust-building strategies and their impact on platforms.

Against this backdrop, this study focuses on China’s medical crowdfunding context, taking trust-building as the entry point to explore consultants’ core trust-building strategies and analyze their impact on platform sustainability. The research questions are: (1) What trust-building strategies do fundraising consultants use in interactions with patients and families? (2) How do these strategies influence the sustainable development of medical crowdfunding platforms? This study first (1) reviews the current research achievements in the field of medical crowdfunding, revealing the contributions and shortcomings of current theoretical research; (2) describes the research methods used in this study; (3) presents the results of data analysis, which are the core strategies for building trust in fundraising consultants. Finally, this study delves into the impact of these strategies on the sustainable development of medical crowdfunding platforms, offering targeted recommendations for optimization and improvement. In summary, by shifting from traditional macrolevel analysis to microlevel interaction scenarios at the service frontline, this study bridges research gaps in trust-building processes to a certain extent and provides theoretical and practical insights for patients and platform managers, promoting industry health and sustainability.

## Methods

### Design

A phenomenological qualitative research design involving in-depth semistructured interviews was chosen. Originating in Europe approximately 60 years ago, phenomenological research is defined as “research that seeks to describe the essence of a phenomenon by exploring it from the perspective of those who have experienced it” [37]. The subjective experiences accumulated by fundraising consultants in their practical work are central to understanding trust construction, and phenomenology is uniquely suited to dissect these experiences from the insiders’ perspective. Thus, qualitative phenomenological research enables researchers to conduct a detailed investigation into the specific strategies used by fundraising consultants to gain the trust of patients and their families, as well as the impact of these strategies on the sustainable development of platforms. The data analysis was based on Colaizzi’s methodology, which has been shown to be rigorous and robust. Colaizzi’s 7-step analytic methodology [38] guided the data analysis process by providing the researcher with detailed and sequential steps to improve the reliability and dependability of the results [39].

### Participants

This study used purposeful sampling to obtain a heterogeneous sample, aiming to explore the specific strategies fundraising consultants use to gain the trust of patients and their families, thereby laying a foundation for subsequent in-depth discussions on how these strategies impact platform sustainability. The sample size was determined as follows: after data saturation was reached, an additional 2 fundraising consultants were interviewed. Recruitment was terminated when no new themes emerged.

All participants were from 4 Chinese internet-based medical crowdfunding platforms. The inclusion criteria were (1) having worked as a fundraising consultant for at least 1 year to ensure sufficient practical experience, (2) engaging in regular direct communication with patients and their families to obtain firsthand experience of trust-building processes, and (3) willingness to participate in in-depth interviews and detailed sharing of work experiences and strategies related to trust-building. The exclusion criteria were (1) individuals who had suspended their role as fundraising consultants in the past 6 months, as prolonged absence from the work context might compromise the timeliness and completeness of their understanding of current trust-building strategies; (2) consultants with language or communication barriers that prevented clear and comprehensive articulation of work experiences to avoid information transmission biases interfering with research analysis; and (3) a diagnosis of severe mental illness.

Sixteen fundraising consultants aged 25-36 years participated in the study. Most were male (n=9), were 30 years old (n=4), were graduated from junior colleges (n=12), and had 4 years of work experience (n=5; Table 1). To ensure confidentiality, participants’ real names were replaced with case numbers.

**Table 1.** Demographic characteristics of fundraising consultants in four Chinese medical crowdfunding platforms (November 2024–March 2025, N=16, phenomenological qualitative study).

Participant identification	Sex	Age (years)	Education level	Work experience (years)
1	Male	25	Junior College	3
2	Male	34	Junior College	3
3	Male	30	Senior High School	2
4	Female	25	Junior College	4
5	Male	29	Junior College	4
6	Female	31	Senior High School	2
7	Female	31	Junior College	5
8	Male	29	Junior College	1
9	Male	36	Junior College	3
10	Male	30	Junior College	4
11	Female	27	Senior High School	2
12	Male	29	Senior High School	4
13	Female	33	Junior College	4
14	Female	30	Junior College	5
15	Male	28	Junior College	2
16	Female	30	Junior College	3

Prior to the final recruitment of 16 participants, a total of 22 fundraising consultants were contacted and preliminarily assessed. Among them, 6 were excluded due to failure to meet the inclusion criteria or meeting the exclusion criteria. The reasons for exclusion are as follows: Four fundraising consultants were excluded because they had suspended their relevant work in the past 6 months. Due to their temporary disengagement from the field of medical crowdfunding practice, their understanding of trust-building strategies might differ from the current reality, making it difficult to provide firsthand experience that closely reflects actual practices.

Two fundraising consultants were excluded due to language expression or communication barriers that prevented them from clearly and accurately describing their work experiences. This study used in-depth semistructured interviews, which highly rely on participants’ ability to articulate detailed implementation of strategies. Communication barriers could compromise the accuracy and reliability of the information collected; thus, these individuals were excluded.

### Data Collection

Data were collected through individual in-depth semistructured interviews, all of which were conducted jointly by the first and second authors. The interviews took place between November 2024 and March 2025, with each session arranged at a time chosen by the participants and led by the first author. A variety of settings were used, including offices, Chinese restaurants, and parks, resulting in a total of 28 interview sessions with 16 participants. The 28 interview sessions had an average duration of 59 minutes (SD 12).

Some participants were interviewed multiple times, primarily to address potential information gaps from the initial interviews and to ensure a comprehensive and in-depth exploration of the trust-building strategies used by fundraising consultants. During the actual data collection process, 8 participants were interviewed once while the other 8 underwent multiple interviews. Specifically, 6 participants were interviewed twice, and 2 participants were interviewed 4 times. These participants were identified as having 2 types of information requiring further supplementation: one pertained to overly generalized descriptions of the practical details of certain strategies, and the other involved relatively cautious disclosures of sensitive information. In light of these circumstances, targeted follow-up questions were used in subsequent interviews to gather more concrete details regarding the implementation of trust-building strategies, thereby minimizing the impact of informational ambiguity on the quality of the study.

At the conclusion of each interview, an open-ended prompt “Do you believe there is any other important information you would like to add?” was used to systematically verify the comprehensiveness and depth of the topics covered. All participants provided similar responses, indicating that “the questions have fully covered the core points.” All interviews were audio-recorded with the written consent of the participants. Verbatim transcription was completed by the first and second authors within 24 hours after each interview, and all personally identifiable information in the transcripts was anonymized. The transcripts were then returned to the participants for review and confirmation of accuracy. No participants withdrew during the interview process, and all interviews were completed in full.

An interview topic guide was also developed (Multimedia Appendix 1) through a rigorous process. First, a preliminary interview outline was drafted based on an extensive review of existing literature. Relevant literature included studies in the field of medical crowdfunding, particularly those related to trust-building, as well as references on qualitative interview methodologies. Subsequently, the guide was rigorously reviewed by a professor (JH), who optimized the logical flow and wording of the questions and added follow-up prompts, such as “Please describe in detail the implementation process and feedback of trust-building strategies with specific examples” to ensure the depth of data collection. Thereafter, pilot interviews were conducted with 2 fundraising consultants who met the study’s inclusion criteria. The purpose was to check the clarity and comprehensibility of the interview questions and to estimate the time required for formal interviews. Data from the pilot interviews were not included in the final analysis. Based on feedback from these pilot interviews, additional prompts, such as “Can you expand on that?” were incorporated to encourage participants to provide more rich and illustrative examples. On this basis, the final interview guide was confirmed.

### Data Analysis

Colaizzi’s methodology for analyzing the narratives of each interview involves seven steps: (1) read the transcript to become familiar with and understand the content of the interviews; (2) locate and extract statements related to trust-building strategy from the transcript; (3) formulate meanings; (4) divide all meanings into categories, theme clusters, and themes; (5) define all emergent themes in an exhaustive description; (6) describe the basic structure of the focal phenomena; and (7) return the results to the participants to ensure their accuracy [40].

The first and second authors analyzed each transcript to conduct the initial analysis. During the data analysis process, reflexivity and multiple validation strategies were adopted. For instance, regular research team meetings were held where each researcher was required to present the rationale behind their analysis. In cases of disagreement among researchers, in-depth discussions were conducted until full consensus was reached. This approach helped mitigate analytical biases that might arise from individual perspectives, thereby enhancing the objectivity of the conclusions. To ensure the reliability of the interpretive analysis, member checking was also implemented. A copy of the analysis was sent to 3 randomly selected participants, who were asked whether their interpretation of the data aligned with our analysis. Participants were invited to clarify their viewpoints if they felt misunderstood. Furthermore, 5 fundraising consultants with profiles similar to those of our participants were invited to review the research findings. They unanimously confirmed that the results accurately reflected their trust-building strategies.

### Ethical Considerations

This study received ethical approval from the Ethics Review Committee of Jiangsu Province Hospital (2025-SR-571). All participants provided written informed consent before interviews, with the consent form clearly explaining the research purpose, procedures, potential risks, rights, and voluntary withdrawal mechanisms. To fully protect participant privacy, personal information, such as names, ages, education levels, and years of work experience was replaced with anonymous codes. All audio recordings were verbatim transcribed within 24 hours, and all identifiers were anonymized in the transcripts. Each participant received monetary compensation after the interview in the form of an electronic gift card valued at 80 Chinese Yuan (approximately US$ 11) as a token of appreciation for their time and valuable insights shared. Moreover, no personally identifiable images, audio, or textual information of participants were used in the paper, supplementary materials, or any public forms of dissemination.

## Results

### Overview

The following three themes emerged from the categorized interview data: (1) establishing initial communication, (2) identifying doubts, and (3) addressing doubts. Each theme is supported by several subthemes, collectively describing the strategies fundraising consultants use to gain the trust of patients and their families (Multimedia Appendix 2).

### Theme 1: Establishing Initial Communication

#### Overview

All participants agreed that the primary step in building trust is initiating preliminary communication with potential users, as this forms the foundation of trust-based relationships. Among the strategies, using skillful scripted openings to start conversations and conveying empathy and care through sincere expressions emerged as 2 core approaches to bridge the distance between consultants and patients and their families.

#### Subtheme 1: Using Scripted Opening Techniques to Initiate Conversations

Scripted techniques play a crucial role in starting dialogues with potential users. Participants typically approached conversations in a natural, friendly manner, gradually guiding patients and their families to open up and share illness-related information. For instance, the consultant in Case 2 mentioned, when sharing his communication approach, that he progressively guides family members of patients to open up by simulating casual daily conversations:

*Every time I encounter a patient or family member who might need crowdfunding, I sit next to them as casually as visiting a neighbor. I start with everyday topics in a gentle, kind tone—like asking, ‘Is someone in your family hospitalized here? How long have they been here?’ It’s just like chatting with a neighbor… If they’re quiet, I don’t rush. I wait patiently, try a few more questions, and slowly encourage them to talk*.

[Case 2]

The consultant in Case 6 placed greater emphasis on understanding the other party’s financial pressure. By inquiring about treatment costs and family burdens, they both gathered information and conveyed care:

*I usually first ask about their current financial situation: how much they’ve spent so far, whether the family is under significant financial pressure. Then I talk with them about the illness and future treatment costs. These questions help me understand their financial reality, and more importantly, let them feel I’m genuinely here to solve problems*.

[Case 6]

Moreover, when the consultant in Case 8 identified that family members of patients had already exhausted all means to borrow money, they would emphasize the practical value of crowdfunding in alleviating debt burdens:

*If I notice the family is already borrowing money everywhere, ie,mphasize how crowdfunding can ease debt pressure. I’ll say, ‘You’ve been borrowing from relatives and friends to treat your family member—it’s really tough. Online crowdfunding could reduce that repayment burden a lot.’*

[Case 8]

The core of this scripted strategy lies not in persuasive tactics but in building rapport through natural, approachable interactions, guiding patients and families to voluntarily share information and reveal needs. Its essence is to transform the consultant’s professional role into that of a relatable problem-solving partner, making crowdfunding services more accessible to the target group.

#### Subtheme 2: Expressing Empathy and Care

Beyond using scripted openings to initiate interactions, establishing emotional resonance with patients and their families is another critical component of initial communication. When patients and their families face illness and financial strain, they endure immense psychological distress and anxiety, making participants’ expressions of empathy and care particularly significant. The consultant in Case 4 mentioned that expressing understanding can effectively bridge the distance:

*When patients or their families share their experiences, I listen attentively and nod occasionally to show I understand. I also share relatable stories, like the hardships my own relatives faced during illness. This helps them feel truly understood, making them more willing to keep talking*.

[Case 4]

The consultant in Case 7 demonstrated care through detailed actions:

*I tell them about similar cases I’ve handled, letting them know they’re not alone—many others have endured the same pain and received help through online crowdfunding. For instance, a patient once cried to me that their family was ruined financially from medical bills. I told them about another family that survived desperation through crowdfunding, to give them hope. Afterward, I’d say, ‘See? Others made it through this. You can too. Let’s find a way together.’ I’d also hand them tissues and ask about their kids’ visits or who’s caring for the patient—little details that show I’m not just reciting stories, but truly grasp their distress and care about their situation.*

[Case 7]

The consultant in Case 1 prioritized providing emotional support over direct promotion when encountering emotionally distressed family members:

*Sometimes I encounter family members crying on hallway benches. I never mention fundraising right away. First, I offer a tissue and ask gently, ‘Ma’am, you’ve been sitting here a while—are you feeling really overwhelmed? My own family dealt with a serious illness before; I know exactly how draining it is…’ Once they calm down a bit, I add, ‘Lots of kind people want to help. Our platform just raised 200,000 yuan for a family with a similar condition—would you like to hear how they did it?’ This approach works better than directly promoting online crowdfunding.*

[Case 1]

In summary, participants used a range of strategies during initial interactions with potential applicants. These strategies not only effectively encouraged patients and their families to open up and share information but also fostered emotional resonance, creating a communicative atmosphere of trust and understanding that paved the way for introducing medical crowdfunding in subsequent conversations.

### Theme 2: Identifying Doubts

#### Overview

After establishing communication with patients and their families, participants often need to directly address the various doubts raised by them. These doubts reflect the cautious attitude of patients and their families toward medical crowdfunding. Only by accurately identifying such questions and concerns can targeted solutions be better formulated, thereby promoting the establishment of trust relationships. Therefore, identifying doubts constitutes a critical link in the trust-building process. In practice, participants found that the common doubts of patients and their families mainly focus on 3 aspects, including the authenticity of consultants’ identities, the standardization of fund operations, and the security of personal information.

#### Subtheme 1: Authenticity of Identity

Patients and their families often doubted the authenticity of participants’ identities, questioning whether they were credible professionals, which made it difficult to establish trust in a short period. Several participants shared their experiences, revealing the vigilance and specific behaviors of patients and their families.

The consultant in Case 9 encountered questioning and defensive behaviors:

*Many patients and their families directly ask, ‘Which organization are you from? Are you here to promote something or cheat money?’ One elderly man even took out his phone to record me, saying, ‘I’ll film this in case you’re a bad person—I need evidence.’ Another time, I met a couple: the husband sat silently with a cold expression, while the wife asked a few questions but finally said, ‘We don’t need this. Don’t try to trick us,’ and drove me away.*

[Case 9]

It is evident that patients and their families generally harbor distrust regarding the authenticity of participants’ identities. As strangers initiating contact, fundraising consultants inherently tend to arouse public suspicion. Moreover, patients’ and families’ past negative experiences with sales pitches or fraud have further deepened their defensive attitudes. Both direct verbal questioning and defensive behaviors reflect their vigilance when interacting with fundraising consultants.

#### Subtheme 2: Standardization of Fund Operations

Patients and their families expressed doubts about the fund operations of medical crowdfunding platforms, which primarily centered on 3 core issues, such as transparency of funding sources, timeliness of fund disbursement, and reasonableness of fees.

The consultant in Case 5 was frequently asked about specific details regarding the receipt of funds:

*Many patients directly ask me: Where does the crowdfunded money come from? Can we really receive it? How long will it take, and what conditions must be met to get the money?*

[Case 5]

The consultant in Case 13 shared a typical case illustrating how information opacity exacerbates patients’ anxiety:

*I once met a woman raising funds for her son’s bone marrow transplant. She clutched her phone and repeatedly asked: ‘You say people are donating, but how do I know who the donors are? When will the money be transferred to my bank card?’ Later, I learned she was so anxious about waiting for the funds for surgery that she couldn’t sleep. However, the platform only showed the fundraising progress without explaining the specific disbursement process, leaving her completely uncertain.*

[Case 13]

Notably, regarding the reasonableness of fees, patients and their families showed particularly strong resistance to platform service charges.

*Many patients ask us: ‘After I launch a crowdfunding campaign, most of the donations come from friends and relatives in my social circle. Since this money is from loved ones, why does your platform charge a fee?’*

[Case 10]

#### Subtheme 3: Security of Personal Information

Patients and their families also expressed strong concerns about the security of their personal information. Since initiating a medical crowdfunding campaign on the platform requires submitting a large amount of sensitive information—such as the patient’s ID number, medical diagnosis, and financial status—patients commonly worried about privacy breaches. Cases shared by participants illustrate their anxiety.

The consultant in Case 6 observed:

*Many patients abandon the online fundraising application when they reach the step of uploading ID cards and other private information, as they fear the platform might use their information for illegal activities.*

[Case 6]

The consultant in Case 3 encountered patients who were extremely sensitive about information exposure:

*An elderly person living alone repeatedly asked, ‘Will this information be accessible to others?’ When I mentioned that some details would be made public, they immediately waved their hand and said, ‘I’d rather not raise money than expose all my family’s details.’*

[Case 3]

Resistance intensified when sensitive information, such as property ownership and financial status, was involved:

*Many patients and their families ask if they can skip providing sensitive details like property ownership, financial assets, or home addresses. They’re truly worried that if this information is made public and accessible to everyone, it might be exploited by criminals.*

[Case 15]

In summary, the doubts of patients and their families regarding medical crowdfunding platforms are systemic and multidimensional. These concerns essentially stem from their dual needs for resources and security amid hardship. They not only reflect deep-seated issues in medical crowdfunding platforms related to trust-building, information transparency, and the reasonableness of rules but also impose higher demands on participants’ communication strategies and platform operating models. Only by addressing these doubts can the concerns of patients and their families be truly alleviated, fostering the sustainable development of medical crowdfunding platforms.

### Theme 3: Addressing Doubts

#### Overview

In response to the aforementioned questions and concerns from patients and their families, participants typically used strategies to enhance trust in medical crowdfunding platforms. Key approaches included presenting valid credentials, leveraging endorsements from partnerships with medical institutions, showcasing successful cases, and offering additional commitments.

#### Subtheme 1: Presenting Valid Credentials

To quickly alleviate doubts about participants’ identities and the platform, presenting valid credentials emerged as the most direct strategy. The consultant in Case 12 typically presents work credentials immediately:

*If patients or their families are skeptical of my identity, I immediately show them my work Identification. Generally, after they carefully review the information on the Identification—including my name, photo, and affiliated platform—their attitude noticeably softens*.

[Case 12]

The consultant in Case 5 shared an example of building trust through detailed verification of credentials:

*I once met someone raising funds for their father, who looked at me with obvious wariness. As soon as I started introducing myself, he stepped back. I promptly took out my work Identification, pointed to the name, photo, and platform logo, and said: ‘Look, this is my work credential, and it even has an anti-counterfeit code.’ He took the Identification, checked it repeatedly, and even scanned the QR code twice. After confirming the information was correct, his tone softened: ‘Sorry, there are so many scammers these days. I figured it’s better to be cautious.’*

[Case 5]

#### Subtheme 2: Leveraging Endorsements From Medical Institutions

Another common strategy used by participants to alleviate patients’ and families’ doubts is building trust through partnerships with authoritative institutions, such as hospitals. The consultant in Case 16 would emphasize the platform’s formal collaboration with medical institutions:

*I tell patients and their families that our platform collaborates with many well-known medical institutions. Patients receiving treatment at these hospitals can launch crowdfunding campaigns through our platform... Endorsements from these partner institutions make patients perceive our platform as highly reliable, leading them to choose us for their fundraising needs...Every time a patient says ‘thank you’, I feel that what I do is truly meaningful. For me, this job is not just about making a living—it also feels like doing a good deed*.

[Case 16]

The consultants in Case 8 and Case 11 enhanced credibility by leveraging physical evidence within hospital settings or authoritative affiliations:

*I take patients and their families to see the promotional posters and cooperation announcements about our platform displayed in hospitals, allowing them to visually confirm our partnership with the hospital.*

[Case 8]

*I explain that I’m visiting on behalf of the hospital to identify patients in financial distress who need fundraising support. People usually lower their guard when they hear I’m commissioned by the hospital.*

[Case 11]

The consultant in Case 14 even established trust by introducing third-party medical staff:

*For patients or families with high vigilance, I invite them to the hospital’s social work office. Ostensibly, I help arrange for medical social workers to assess their eligibility for hospital-based medical assistance; in reality, this lets them observe my rapport with doctors in white coats, which strengthens their trust... Some patients and families later told me, ‘If the hospital approves of you, we have no reason to worry.’*

[Case 14]

#### Subtheme 3: Displaying Successful Cases

Sharing stories of successful fundraising experiences from patients with similar circumstances is a key strategy to motivate potential users. Participants typically select real cases with comparable illness severity and financial situations, using detailed narratives and visual data to help potential users perceive that they too could resolve part of their funding issues through the medical crowdfunding platform.

The consultant in Case 1 provided hope to patients by showcasing successful cases of similar medical conditions:

*I show patients successful crowdfunding cases of people with similar illnesses, detailing their fundraising process and the final amount raised. For example, a few days ago, I met a mother whose child needed treatment for congenital heart disease. She was in tears, despairing that she could never afford the surgery. I immediately pulled up cases of patients with the same condition on my phone, showing her how some had raised significant funds through the platform. After seeing them, she seemed to find hope and said she wanted to try…That feeling of helping patients find hope has made me realize that this job is not just about making a living; it’s about making a tangible difference in people’s lives. It makes me feel that my work is truly valuable.*

[Case 1]

It is evident that when patients witness others with the same illness and hardships regaining hope through the crowdfunding platform and hear firsthand accounts of transformation from despair to hope, the abstract concept of crowdfunding is transformed into tangible hope. This strategy not only alleviates patients’ and families’ concerns but also helps them tangibly recognize the feasibility and effectiveness of medical crowdfunding, thereby encouraging them to proactively seek support from the platform.

#### Subtheme 4: Promising Additional Conditions

Participants strengthen their identification with patients and their families by implementing additional commitment strategies, thereby building trust relationships. For example, committing to protecting personal information security, assisting with fundraising processes, and helping patients apply for economic subsidies.

The consultant in Case 3 admitted that, even though the platform could not fully guarantee information security, they would still make commitments to gain trust:

*Honestly, our platform can’t fully protect users’ information security—after all, the information is public and accessible to anyone. But to get them to agree to launch the fundraiser, I tell them the platform will protect their personal information, and that I’ll help resolve any issues throughout the process... In short, I do my best to show I’m fully responsible, which makes patients more willing to trust me with their case.*

[Case 3]

Notably, some participants engaged in inappropriate promising practices. When patients or families requested help with financial subsidy applications, certain participants would promise assistance regardless of eligibility, only to later dismiss the request with “ineligibility” after the crowdfunding campaign was launched. The consultant in Case 10 admitted that due to performance pressure, they would promise financial subsidies that could not actually be delivered:

*When patients ask if I can help them apply for other financial subsidies after fundraising, I usually agree first. In fact, I have a rough idea whether they qualify for medical assistance—even if some don’t, I still promise to help. This makes them feel I’m doing my best, which increases their trust in me…I am aware that this approach is less than ideal, but the pressure from performance assessments is substantial. If I fail to meet the targets, my income will be affected. Therefore, I sometimes resort to this method to secure their trust*.

[Case 10]

Even more concerning, with particularly difficult-to-communicate patients or families, some participants would deceive them with promises of additional benefits to gain trust, only to later avoid responsibility by claiming application failure*.*

*If patients or families are hard to communicate with, I first trick them by saying I can help apply for other benefits. Once they launch the crowdfunding, I tell them they’re ineligible or the application failed. As long as the patient eventually initiates the crowdfunding campaign, my work targets are considered fulfilled.*

[Case 14]

In summary, participants systematically enhanced patients’ and families’ trust in medical crowdfunding platforms through strategies, including presenting valid credentials to verify legitimacy, leveraging hospital partnerships for trust endorsements, sharing successful cases of similar patients, and making supplementary commitments. However, ethical misconduct in commitment strategies—though potentially boosting short-term conversion rates—carries risks of eroding industry credibility, reflecting a deep-seated conflict between commercial logic and service ethics in the crowdfunding ecosystem.

## Discussion

### Principal Findings

This study found that consultants’ trust-building strategies exhibit a clear binary differentiation feature, characterized by the coexistence of positive and negative strategies. Positive strategies, such as empathy-based communication techniques, presenting valid credentials, leveraging endorsements from medical institution partnerships, and displaying successful cases, are stable and sustainable. They help platforms accumulate long-term reputations and effectively break down patients’ trust barriers. Negative strategies, by contrast, manifest as unethical behaviors, such as false promises and inducements. While these may temporarily improve conversion rates and boost the number of fundraising campaigns launched, they undermine platform credibility to some extent and are detrimental to long-term healthy development.

These findings can be further elucidated through relevant theories of trust construction. First, the core of trust-building lies in transforming an unfamiliar relationship into interpersonal trust. To achieve this, fundraising consultants strategically leverage patients’ inherent institutional trust in the health care system. Behaviors, such as displaying cooperative agreements with hospitals or guiding patients to medical social work offices, essentially use a trust transfer mechanism to facilitate this conversion. According to this theory, an individual’s institutional trust in authoritative organizations can be transferred across contexts to associated entities; that is, trust can migrate from a trusted object to an unfamiliar one [41]. In China, doctors and hospitals, as symbols of authority, enjoy a high degree of trust among patients and their families [42]. This profound institutional trust provides a solid social foundation for trust transfer. By demonstrating their connection to trusted medical institutions, fundraising consultants successfully transfer patients’ institutional trust to themselves (interpersonal trust) and the platform they represent, thereby reducing patients’ cognitive uncertainty and skepticism [43].

Second, behaviors shown by consultants—such as empathetic communication, active listening, sharing personal experiences, offering tissues, and showing care—transcend mere communication techniques and can be viewed as a form of deep emotional labor. By managing their own emotional expressions and adhering to culturally expected patterns of interaction, they strive to establish emotional bonding with patients and families [44]. This type of emotional labor is particularly crucial within a collectivist culture that emphasizes interpersonal harmony and empathetic interaction, and its effectiveness is deeply rooted in this cultural soil. China has a long tradition of valuing harmony within groups [45] and promotes empathic interaction [46], stressing the importance of understanding and care in building trust. Within this context, emotional expression surpasses purely professional behavior and becomes a necessary labor within a specific cultural setting. Consequently, the emotional strategies of fundraising consultants exhibit distinct localized characteristics. These strategies not only fulfill the emotional needs of patients and families, making them feel respected and cared for as whole persons rather than just medical cases [47], but also lay the groundwork for interpersonal trust, ultimately prompting patients to decide to initiate fundraising.

In summary, the trust-building process undertaken by fundraising consultants entails transferring institutional trust and cultivating interpersonal relationships. They transfer the patients’ institutional trust in the health care system to themselves and their platform and then nurture and consolidate interpersonal trust through emotional labor. This mechanism not only reveals the internal logic of trust establishment but also provides a profound basis for understanding how positive strategies contribute to the sustainable development of the platform.

The core of participants’ use of positive strategies lies in influencing the decision-making behaviors of patients and their families by altering their cognitive and emotional states. Specifically, first, at the cognitive level, participants primarily achieved cognitive reconstruction through a trust transfer mechanism. For example, identity-visualizing methods, such as presenting work credentials with anticounterfeit features and displaying partnership announcements with hospitals, allowed patients and their families to visually verify the authenticity of their identities, thereby reducing wariness toward strangers. Second, at the emotional level, participants engaged in emotional labor, such as demonstrating empathy and care and sharing success stories to narrow the emotional distance with patients and their families and evoke hope that their predicament can be resolved. In the context of this study, when patients and their families heard about successful crowdfunding cases, the abstract possibility of crowdfunding success in their minds often transformed into tangible hope. This shift not only helped alleviate their anxiety but also improved their emotional attitudes toward participants and the platform, even enhancing their trust in the effectiveness of crowdfunding, thereby increasing their willingness to proactively launch campaigns on the platform and forming a sustained driving force for platform operations. Scholars have noted that crowdfunding often serves as a resource for both instrumental and emotional social support [48]. This study further finds that the emotional strategies used by fundraising consultants elevate medical crowdfunding beyond mere fundraising to an interactive process of emotional support. This emotional interaction not only validates previous research conclusions but also reveals a deeper logic. When patients and their families gain hope and emotional satisfaction through participants’ emotional strategies, their proactive crowdfunding behavior becomes a critical link connecting individual trust and platform ecology, ultimately forming the emotional foundation for the sustainable development of medical crowdfunding platforms.

In addition to positive strategies, some participants also adopted negative strategies when building trust relationships with patients and their families. Their motivations can be attributed to 2 main factors. First, commercialized performance evaluation constitutes a direct driving force. In modern organizational management systems, it has become common for employees to face performance pressure [49]. Pay for performance, as an important means of motivating employees, has been shown to positively impact work performance [50]. However, while performance pressure drives desirable outcomes, it also entails risks and uncertainties [51]. For example, participants in this study tended to adopt unethical behavioral strategies to achieve performance goals. Second, the vulnerability of vulnerable groups is another key factor that cannot be ignored. When patients experience severe illnesses, they not only face uncertainty, anxiety, and fear from deteriorating health or even the threat of death but also bear heavy financial pressure in most families [52]. This situation plunges them into a state of high vulnerability [53], making them urgently dependent on external assistance [54]. Through experience accumulated over long-term work, participants accurately grasped the vulnerability characteristics and core needs of patient groups and then implemented strategies, such as false inducements to motivate patients and their families to launch medical fundraising. The essence of this behavior is the exploitation of the vulnerability of vulnerable groups, as individuals in helpless situations are more likely to believe seemingly reliable promises. This study not only confirms the vulnerability and dependence of patients as vulnerable groups [53] but also reveals how these characteristics are alienated into a tool to achieve commercial goals.

Through an in-depth analysis of fundraising consultants’ trust-building strategies, this study reveals that their strategic choices are not random but are driven by factors, such as external performance pressure and internal professional identity. Specifically, when consultants face high performance pressure, they tend to adopt negative strategies. This occurs because an excessive emphasis on fundraising targets and commercial returns by platforms effectively marginalizes ethical considerations, prompting consultants to prioritize short-term fundraising goals and consequently choose strategies that may yield immediate results but potentially compromise long-term trust. Conversely, when consultants possess a stronger professional identity or operate within an organizational culture that values service quality and social reputation, they are more inclined to adopt positive strategies. These strategies primarily rely on trust transfer mechanisms, leveraging the established credibility of institutions, such as hospitals, to build sustainable trust relationships.

Based on these findings, this study proposes that fundraising consultants’ trust-building strategies essentially represent a negotiation and balance between commercial performance and helping ethics. This theoretical perspective connects microlevel individual behaviors with macrolevel institutional and cultural contexts, not only explaining why and when consultants adopt different strategies but also providing an analytical framework for understanding the ethical tensions in the commercialization of public services within the Chinese context.

It is also noteworthy that this study identified a concerning phenomenon. Patients and their families expressed strong doubts about platform fund operations and information security, but participants’ responses were generally vague. This interaction pattern of avoiding core issues essentially reflects management deficiencies in the platforms’ fund and information security. Existing research indicates that an organization’s internal management capacity is crucial to its development [55], and organizations with strong management awareness are more likely to achieve better performance and growth [56]. In particular, the visibility and security of data in financial transactions [57] form the foundation of user trust. Vulnerabilities in these areas not only directly harm users’ property security and privacy rights but may also damage the organization’s trustworthiness and reputation [58], exerting a destructive impact on organizational operations and sustainable development.

Based on the identified negative consultant strategies and platform management deficiencies, this study proposes 3 recommendations to enhance platform sustainability and their role in medical assistance. First, implement comprehensive ethics training for fundraising consultants to improve professionalism. Second, strengthen platform oversight with transparent fund management, robust data security, and strict penalties for misconduct. Third, establish a clear legal framework defining ethical standards, fund handling procedures, and privacy protections to guide industry standardization.

The novelty of this study lies in its microlevel perspective on fundraising consultants—a key yet understudied actor—and its contextualization within China’s sociocultural environment, enriching trust construction theory in non-Western settings. Theoretically, it extends trust transfer and emotional labor theories to medical crowdfunding, offering new Chinese case evidence. Practically, it provides actionable insights for platform optimization, industry regulation, and patient decision-making, ultimately enhancing service credibility and equity.

### Limitations

This study has several limitations. First, the inclusion of fundraising consultants from only 4 Chinese platforms limits the generalizability of the findings to other contexts. Second, some interviews were conducted in public settings, such as restaurants and parks, which, despite efforts to select quieter spots, may have influenced participants’ openness, particularly on sensitive topics. Third, the sole focus on consultants—without incorporating views from patients, families, or platform managers—may offer a partial perspective on trust-building and platform operations. Additionally, the absence of quantitative metrics, such as fundraising success rates or patient satisfaction, precludes assessment of the strategies’ practical effectiveness, shifting the emphasis instead to their content and implementation processes. These limitations should be considered when interpreting the results.

### Future Research

This study highlights several avenues for future inquiry. First, research could explore emotional labor competencies among fundraising consultants to develop culturally grounded support guidelines aligned with China’s collectivist context. Second, examining technical and managerial solutions—such as blockchain applications—to enhance fund traceability and data security represents another critical direction. Third, future work should investigate how performance metrics shape consultants’ ethical decision-making. Finally, incorporating perspectives from patients and families would provide a more comprehensive understanding of trust-building effectiveness and help optimize service delivery.

### Conclusions

This study uses a phenomenological approach to explore the core strategies used by fundraising consultants to gain the trust of patients and their families in the context of Chinese medical crowdfunding and further analyzes the impact of these strategies on the sustainable development of medical crowdfunding platforms. The findings reveal that consultants’ trust-building strategies exhibit a dualistic character. On one hand, positive strategies, such as empathetic communication techniques, presenting valid credentials, leveraging endorsements from medical institution partnerships, and showcasing successful cases, effectively break down patients’ trust barriers, accumulate long-term reputations for platforms, and generate positive momentum for healthy development. On the other hand, some consultants adopt unethical negative strategies, including false promises and inducements. While these may temporarily increase the number of fundraising campaigns launched, they severely damage platform credibility in the long term and pose hidden risks to sustainable development. In light of these findings, this study proposes several optimization recommendations, such as strengthening professional ethics training for fundraising consultants, improving platform management, supervision, and punishment systems, and accelerating legislation targeting the online medical crowdfunding sector. These measures will not only enhance public trust in platforms but also promote the standardized and sustainable development of medical crowdfunding platforms, ultimately building a reliable social support network for patients in need.

It is important to clarify that the conclusions of this study are most applicable to contexts that share similar cultural backgrounds and platform operational models with China. Specifically, the boundary conditions can be summarized into 4 key aspects. First, contexts characterized by a collectivist cultural tradition, which emphasizes interpersonal harmony and empathetic interaction, are closely related to the emotional labor strategies adopted by fundraising consultants. Second, the presence of a high level of institutional trust in medical institutions and doctors provides a social foundation for strategies, such as leveraging hospital endorsements. Third, a public medical security system that is still developing, with certain coverage gaps, leads to a higher dependence on crowdfunding among patient families. Fourth, the dominant operational model of medical crowdfunding platforms integrates online and offline approaches, where the offline promotion and involvement of fundraising consultants serve as a core channel for reaching users, rather than a purely online self-initiated model. These factors collectively define the boundary conditions within which the findings of this study are applicable.

## Supplementary material

10.2196/80299 Multimedia Appendix 1 Interview topic guide.

10.2196/80299 Multimedia Appendix 2 Themes and subthemes of trust-building strategies used by fundraising consultants in Chinese medical crowdfunding platforms.

10.2196/80299 Checklist 1 COREQ checklist.
