# Supplementary material for: Trust-Building Strategies of Fundraising Consultants in Chinese Medical Crowdfunding Platforms: Qualitative Study
Source: J Med Internet Res. 2025 Nov 19;27:e80299. doi: 10.2196/80299 (PMC12629622; doi:10.2196/80299)
Supplement: Multimedia Appendix 1 [file jmir-v27-e80299-s001.docx]

**Appendix 1. Interview topic guide**

1. Could you first introduce your years of working as a fundraising consultant on the platform and your main responsibilities?

2. What methods or techniques do you primarily use to establish initial trust when first interacting with patients and their families?

3. What are the most common concerns or doubts expressed by patients and their families regarding medical crowdfunding platforms?

4. In the face of negative public opinion and trust crises surrounding the platform, what strategies have you adopted to rebuild trust with patients and their families? Please describe in detail the implementation process and feedback of trust-building strategies with specific examples.

5. In your opinion, which strategies are most effective in gaining patients’ trust? Why?

6. Is there any case that left a particularly deep impression on you? How did you handle it at the time?

7. Have you ever encountered situations where strategies adopted to meet performance targets conflicted with ethical principles? Please provide an example.

**General Prompts:**

Can you tell me more about that？

Can you expand on that?

Can you think of an example?
