# Supplementary material for: Trust-Building Strategies of Fundraising Consultants in Chinese Medical Crowdfunding Platforms: Qualitative Study
Source: J Med Internet Res. 2025 Nov 19;27:e80299. doi: 10.2196/80299 (PMC12629622; doi:10.2196/80299)
Supplement: Multimedia Appendix 2 [file jmir-v27-e80299-s002.docx]

**Appendix 2. Themes and Subthemes of Trust-Building Strategies Used by Fundraising Consultants in Chinese Medical Crowdfunding Platforms (November 2024–March 2025, Based on In-Depth Semi-Structured Interviews with 16 Consultants)**

| **Theme** | **Subtheme** |
| --- | --- |
| establishing initial communication | using scripted opening techniques to initiate conversations |
|  | expressing empathy and care |
| identifying doubts | authenticity of identity |
|  | standardization of fund operations |
|  | security of personal information |
| addressing doubts | presenting valid credentials |
|  | leveraging endorsements from medical institutions |
|  | displaying successful cases |
|  | promising additional conditions |
