# Supplementary material for: Trust-Building Strategies of Fundraising Consultants in Chinese Medical Crowdfunding Platforms: Qualitative Study
Source: J Med Internet Res. 2025 Nov 19;27:e80299. doi: 10.2196/80299 (PMC12629622; doi:10.2196/80299)
Supplement: Checklist 1 [file jmir-v27-e80299-s003.pdf]

COREQ checklist.

Item No	Guide Questions/Description	Comment
**Domain 1: Research team and reflexivity**		
**Personal Characteristics**		
1. Interviewer/ facilitator	Which author/s conducted the interviews?	QL and JYH conducted the semi-structured interviews.
2. Credentials	What were the researcher’s credentials?	QL holds a Master of Social Work (MSW) degree and is currently a doctoral candidate in Sociology. JYH holds a Doctor of Management degree and works as a professor in the field of Sociology. YTT is a doctoral candidate in Sociology, specializing in research related to the relationships between patients and healthcare workers. LND holds a Doctor of Sociology degree and possesses professional expertise in healthcare. TFL holds a Master of Arts (MA) degree and is currently a doctoral candidate in Sociology.
3. Occupation	What was their occupation at the time of the study?	JYH serves as a professor at the School of Public Administration, Hohai University, and also holds the position of Director of the Population Research Institute at Hohai University. LND acts as the Director of the External Cooperation Department of Jiangsu Provincial People's Hospital. QL, YTT, and TFL are doctoral candidates (pursuing their PhD degrees).
4. Gender	Was the researcher male or female?	Four female researchers and one male researcher.
5. Experience and training	What experience or training did the researchers have?	QL, JYH, YTT, LND, and TFL have extensive experience in conducting qualitative health research. In addition, QL and JYH have published papers related to patients. JYH teaches research methods at the School of Public Administration, Hohai University, where he/she specializes in sociology.
**Relationship with participants**		
6. Relationship established	Was a relationship established prior to study commencement?	No. Everyone is regarded as unbiased researchers, rather than individuals with specific interests, positions, or opinions on the results.

7. Participant knowledge of the interviewer	What did the participants know about the researcher?	The interviewer introduces themselves to the participants, describes the background, scope and purpose of the research, and elaborates on each component of the study. The content adheres to the accurate guidelines approved by the Institutional Review Board (IRB) of all institutions involved in human research ethics review.
8. Interviewer characteristics	What characteristics were reported about the interviewer/facilitator?	Participants are informed of the interviewer's educational background, affiliated institution, and research questions, which serve as the context for the interview. The introduction emphasizes the interviewer's research intention to explore the relevant issue, while refraining from disclosing any specific pre-existing hypotheses or potential biases. The entire process strictly adheres to the guidelines approved by the Institutional Review Boards (IRBs) of all relevant institutions.
**Domain 2: study design**		
**Theoretical framework**		
9. Methodological orientation and Theory	What methodological orientation was stated to underpin the study?	The study was explicitly stated to be underpinned by a phenomenological qualitative research design. This is articulated in the 'Method' section as follows: "A phenomenological qualitative research design involving in-depth semi-structured interviews was chosen."
**Participant selection**		
10. Sampling	How were participants selected?	Purposeful sampling was used. As stated in the manuscript: "Purposeful sampling was used to obtain a heterogeneous sample."
11. Method of approach	How were participants approached?	Send invitations via email to individuals who may meet the following inclusion criteria: (1) having worked as a fundraising consultant for at least one year to ensure sufficient practical experience; (2) engaging in regular direct communication with patients and their families to obtain first-hand experience of trust-building processes; (3) willingness to participate in in-depth interviews and detailed sharing of work experiences and strategies related to trust-building. The recruitment process follows accurate guidelines certified by all human research ethics review committees involved in the institution.
12. Sample size	How many participants were in the study?	16 participants. For detailed information on the demographic data of the study participants, please refer to Table 1.

13. Non-participation Setting	How many people refused to participate or dropped out? Reasons?	Six fundraising consultants who were initially contacted were excluded from the study. Four were excluded because they had suspended their relevant work in the past six months, and two were excluded due to language expression or communication barriers. No participants withdrew during the interview process.
14. Setting of data collection	Where was the data collected?	Data were collected in "a variety of settings, including offices, Chinese restaurants, and parks."
15. Presence of nonparticipants	Was anyone else present besides the participants and researchers?	No
16. Description of sample	What are the important characteristics of the sample?	Fully presented in Table 1.
**Data collection**		
17. Interview guide	Were questions, prompts, and guides provided by the authors? Was it pilot tested?	Yes, an interview topic guide was developed by the authors through a rigorous process that included a literature review, expert review by a professor, and pilot testing with two fundraising consultants. Data from the pilot interviews were not included in the final analysis. Please refer to Appendix 1.
18. Repeat interviews	Were repeat interviews carried out? If yes, how many?	Yes, repeat interviews were carried out. Some participants were interviewed more than once, resulting in a total of 28 interview sessions with 16 participants. Eight participants underwent multiple interviews, with six individuals receiving two interviews and two others receiving four interviews.
19. Audio/visual recording	Did the research use audio or visual recording to collect the data?	Yes, all interviews were audio-recorded with the written consent of the participants.
20. Field notes	Were field notes made during and/or after the interview or focus group?	Following each interview, the interviewer documented brief notes highlighting the interview's nature and key themes emphasized by the interviewee.
21. Duration	What was the duration of the interviews or focus group?	The 28 interview sessions had an average duration of 59 minutes.
22. Data saturation	Was data saturation discussed?	Yes.

23. Transcripts returned	Were transcripts returned to participants for comment and/or correction?	Yes.
**Domain 3: analysis and findings**		
**Data analysis**		
24. Number of data coders	How many data coders coded the data?	The first and second authors analyzed each transcript to conduct the initial analysis.
25. Description of the coding tree	Did the authors provide a description of the coding tree?	Yes. The authors provided a description of the thematic structure, presenting "three main themes and nine subthemes" that emerged from the data analysis, which constitutes the coding tree.
26. Derivation of themes	Were themes identified in advance or derived from the data?	Derived from the data.
27. Software	What software, if applicable, was used to manage the data?	We did not use specialized qualitative data analysis software for coding, primarily for the following two reasons: First, the scale of interview data in this study was relatively limited, making the entire textual content suitable for manual processing. Second, during our repeated reviews of the raw materials, we found that manual coding helped maintain sensitivity to context and facilitated the capture of new insights and emerging themes based on a deeper understanding. Therefore, we ultimately chose to complete the coding work manually to ensure closer alignment with the data itself and to maintain flexibility in the analytical process.<br>We are also aware that manual coding may have certain limitations. Nonetheless, in this study, we endeavored to ensure the rigor and transparency of the analytical process through team discussions and multiple rounds of review.
28. Participant checking	Did participants provide feedback on the findings?	Yes. Member checking was also implemented. A copy of the analysis was sent to three randomly selected participants, who were asked to confirm whether the interpretation aligned with their experiences.
**Reporting**		

29. Quotations presented	Were participant quotations presented to illustrate the themes/findings? Was each quotation identified?	Yes. Numerous participant quotations were presented throughout the results section, and each quotation was clearly identified with its corresponding participant case number.
30. Data and consistent findings	Was there consistency between the data presented and the findings?	Yes. There is clear consistency as all findings are systematically supported by direct participant quotations and detailed descriptions derived from the interview data, with analytical interpretations consistently linked to the presented evidence.
31. Clarity of major themes	Were major themes clearly presented in the findings?	Yes. The findings section clearly presents "three main themes and nine subthemes" that emerged from the data analysis, with each theme systematically described and supported by participant quotations.
32. Clarity of minor themes	Is there a description of diverse cases or a discussion of minor themes?	The findings present diverse participant perspectives through multiple identified case examples, but do not include a specific discussion of minor themes separate from the main thematic structure.
